# Targeted Urine Metabolomics for Monitoring Renal Allograft Injury and Immunosuppression in Pediatric Patients

**DOI:** 10.3390/jcm9082341

**Published:** 2020-07-22

**Authors:** Tara K. Sigdel, Andrew W. Schroeder, Joshua Y. C. Yang, Reuben D. Sarwal, Juliane M. Liberto, Minnie M. Sarwal

**Affiliations:** Division of Transplant Surgery, Department of Surgery, University of California San Francisco, San Francisco, CA 94143, USA; tara.sigdel@ucsf.edu (T.K.S.); andrew.schroeder@ucsf.edu (A.W.S.); joshua.yang@alumni.ucsf.edu (J.Y.C.Y.); reuben.sarwal@ucsf.edu (R.D.S.); jlibert7@jhmi.edu (J.M.L.)

**Keywords:** kidney transplantation, metabolomics, immunosuppression, urine, acute rejection, immunosuppression, allograft

## Abstract

Despite new advancements in surgical tools and therapies, exposure to immunosuppressive drugs related to non-immune and immune injuries can cause slow deterioration and premature failure of organ transplants. Diagnosis of these injuries by non-invasive urine monitoring would be a significant clinical advancement for patient management, especially in pediatric cohorts. We investigated the metabolomic profiles of biopsy matched urine samples from 310 unique kidney transplant recipients using gas chromatography–mass spectrometry (GC-MS). Focused metabolite panels were identified that could detect biopsy confirmed acute rejection with 92.9% sensitivity and 96.3% specificity (11 metabolites) and could differentiate BK viral nephritis (BKVN) from acute rejection with 88.9% sensitivity and 94.8% specificity (4 metabolites). Overall, targeted metabolomic analyses of biopsy-matched urine samples enabled the generation of refined metabolite panels that non-invasively detect graft injury phenotypes with high confidence. These urine biomarkers can be rapidly assessed for non-invasive diagnosis of specific transplant injuries, opening the window for precision transplant medicine.

## 1. Introduction

Kidney transplantation (KTx) is the preferred method of treatment for end-stage kidney failure [1]. Increasing the longevity of transplanted kidneys is critical because of the shortage of available kidneys and kidney donors [2]. While improved short-term survival of the transplanted kidney has been attributed to better immunosuppressive drugs and sophistication in organ procurement and surgical methods [3], long-term survival outcomes have largely remained limited and unchanged [4]. Currently used methods of KTx monitoring, such as patient serum creatinine and proteinuria, are neither sufficiently sensitive nor specific to detect early-stage injury and only detect advanced and often irreversible tissue injury [5]. Additionally, kidney biopsies cannot easily be used to predict injury [6,7]. Over recent years, the application of high throughput technologies towards a more discovery-based approach for correlative biomarkers of graft injury have utilized sequencing [8] gene expression, proteomic [9,10,11,12,13,14], and metabolomic methods [15,16]. Many of these approaches show background signals of other clinical confounders, such as immunosuppression exposure [17], and thus require the application of more customized and robust analytical techniques for improving the diagnostic accuracy of biomarkers in blood and urine to reflect different transplant (Tx) injury phenotypes [18,19,20].

In this study, we hypothesized that the recipient’s immune response towards the graft induces immunological and downstream metabolic changes at the time of specific injuries, such as acute rejection (AR), which result in perturbations in specific urine metabolite concentrations. We also hypothesized that specific metabolic pathways are injury-specific such that a panel of metabolites can be used as a surrogate biomarker to monitor KTx injuries. In this report, we present our findings from a comprehensive targeted metabolomics analysis of urine collected from pediatric KTx patients. These samples have been biopsy matched, providing an accurate phenotype characterization, and enabling exploration of metabolic pathways associated with KTx dysfunction.

## 2. Experimental Section

### 2.1. Patients and Samples

Biobanked urine samples available in the Sarwal lab from previously funded studies were screened for matching biopsy data on the day of urine collection. Out of a total of 2016 biobanked urine samples collected between 2006 and 2009, 770 were biopsy-matched, of which 326 unique and clinically annotated urine samples were included in the first part of this study. These patients were on calcineurin inhibitor (CNI) based immunosuppression (IS). All urine samples were stored at −80 °C with urine processing techniques, procedures, and conditions in which we have previously shown negligible degradation of urine components [11].

All samples from our biobank were matched with transplant biopsies; all biopsies were read by a central pathologist and scored by the Banff and Chronic Allograft Damage Index (CADI) [21,22,23] as acute cellular or humoral rejection with clinical graft dysfunction, and tubulitis and/or vasculitis on histology (AR; *n* = 106) [24], stable with no histological or clinical graft injury (stable graft function (STA); *n* = 111), interstitial fibrosis and tubular atrophy (IFTA; *n* = 71) [25], and BK viral nephritis with SV40 staining on histology, with/without clinical graft dysfunction (BK viral nephritis (BKVN); *n* = 22). Intragraft C4d stains were performed to assess for antibody-mediated rejection (ABMR). AR was defined, at minimum, by the following criteria: (i) TCMR consisting of either a tubulitis (t) score > 2 accompanied by an interstitial inflammation score > 2 or vascular changes (v) score > 0; (ii) C4d-positive ABMR consisting of positive donor-specific antibodies (DSAs) with a glomerulitis (g) score > 0 or peritubular capillaritis score (ptc) > 0 or v > 0 with unexplained acute tubular necrosis/thrombotic microangiopathy (ATN/TMA) with C4d = 2; or (iii) C4d-negative ABMR consisting of positive DSA with unexplained ATN/TMA with g + ptc ≥ 2 and C4d = 0 or 1. Stable allografts were defined by an absence of substantial injury on the matched biopsy pathology and definitions of the inflammation or i score and the tubulitis or t score. IFTA used standard pathology definitions as described by the Banff schema on the paired biopsies from each individual urine sample.

This study was conducted in accordance with the relevant guidelines and regulations as approved by the University of California San Francisco (UCSF) Human Research Protection Program Institutional Review Board (IRB) under IRB #14-13573. All patients provided written informed consent. In cases of pediatric and young adult patients, written informed consent was obtained from a parent and/or legal guardian to participate in the research, in full adherence to the Declaration of Helsinki. The clinical and research activities being reported are consistent with the Principles of the Declaration of Istanbul as outlined in the ‘Declaration of Istanbul on Organ Trafficking and Transplantation Tourism’. As such, no organs or tissues were procured from prisoners. All organs or tissues were procured from the Departments of Surgery at either UCSF or Stanford University.

### 2.2. Urine Collection, Initial Processing, Storage, and GC/MS-TOF Analysis

Second morning void mid-stream urine (50–100 mL) was collected in sterile containers and was centrifuged at 2000× *g* for 20 min at room temperature within 1 h of collection. Specifically, the urine specimens were collected in sterile polypropylene collection tubes that are leak-resistant with a sterility seal. Processing of the urine was done all in one bath with sterile polypropylene plastic tubes. The supernatant was separated from the pellet containing any particulate matter including cells and cell debris. The pH of the supernatant was adjusted to 7.0 with Tris-HCL and stored at −80 °C in polypropylene plastic tubes until further analysis. The identification of metabolites followed the well-established FiehnLib protocol [26]. In brief, all metabolite reference standards underwent a two-step derivatization procedure following the previously published protocol [27]. The derivatization of urine metabolites procedure has been described previously [27]. Briefly, neat urine samples were lyophilized without further pretreatment after our initial finding of severe alterations using urease treatments. To the dried samples, 20 µL of 40 mg/mL methoxylamine hydrochloride in pyridine was added, and samples were agitated at 30 °C for 30 min. Subsequently, 180 µL of trimethylsilylating agent *N*-methyl-*N*-trimethylsilyltrifluoroacetamide (MSTFA) was added, and samples were agitated at 37 °C for 30 min. GC–MS analysis was performed using an Agilent 6890 N gas chromatograph (Atlanta, GA, USA) interfaced to a time-of-flight (TOF) Pegasus III mass spectrometer (Leco, St. Joseph, MI, USA) [27]. Automated injections were performed with a programmable robotic Gerstel MPS2 multipurpose sampler (Mülheim an der Ruhr, Germany). The GC was fitted with both an Agilent injector and a Gerstel temperature-programmed injector, cooled injection system (model CIS 4), with a Peltier cooling source. An automated liner exchange (ALEX) designed by Gerstel was used to eliminate cross-contamination from sample matrix occurring between sample runs. Multiple baffled liners for the GC inlet were deactivated with 1 µL injections of MSTFA. The Agilent injector temperature was held constant at 250 °C while the Gerstel injector was programmed (initial temperature 50 °C, hold 0.1 min, and increased at a rate of 10 °C/s to a final temperature of 330 °C, hold time 10 min). Injections of 1 µL were made in split (1:5) mode (purge time 120 s, purge flow 40 mL/min). Chromatography was performed on a Rtx-5Sil MS column (30 m × 0.25 mm inner diameter (i.d.), 0.25 µm film thickness) with an Integra-Guard column (Restek, Bellefonte, PA, USA). Helium carrier gas was used at a constant flow of 1 mL/min. The GC oven temperature program was initially 50 °C with a 1-min hold time and ramping at 20 °C/min to a final temperature of 330 °C with a 5-min hold time before cool-down for a 20 min run time. MS parameters were based on Autotune using FC43 (Perfluorotributylamine) with manufacturer-specific tune settings. Transfer line temperature was 250 °C and electron impact ionization was set at 70 eV. Filament source temperature was at 250 °C and TOF at room temperature. After a solvent delay of 350 s, mass spectra were acquired at 20 scans/s with a mass range of 50 to 500 *m/z*. Initial peak detection and mass spectrum deconvolution were performed with Leco Chroma-TOF software (version 2.25, Leco) and samples were exported to the netCDF format for further data evaluation with MZmine [28] and XCMS [29].

### 2.3. Raw Data Processing and Statistics

All chromatograms were assessed in the same manner by software packages MZmine [28] and XCMS [29]. These packages performed peak finding in an automated and unbiased way using the common MS netCDF file format that enables a unique way of data export irrespective of different instrument platforms. For the raw GC–MS data, the netCDF export function from the Leco ChromaTOF software was used. For MZmine, the *m/z* bin size was set to 0.01, the chromatographic threshold level was set to 0.5, the absolute intensity threshold was set to 2500, the tolerance in *m/z* values was set to 0.5, the tolerance in intensity was set to 1.0, and the minimum peak length was set to 2 s.

The raw data was normalized using urine creatinine, as an internal control, measured as a part of urine metabolome assessment and quantile normalization for batch correction. Moreover, 310 biopsy-matched urine samples, with resulting panels of 266 metabolites, were used for the analyses of both post-transplant injury classification and significant metabolite selection. Non-parametric imputation was applied to these samples via the missForest algorithm [30]. If more there was missing data on more than one-third of the metabolites, these samples were excluded. Sixteen samples met this criterion.

Clustering was performed and visualized with Morpheus (Broad Institute) using average linkage hierarchical clustering. The log-transformed data was median centered, per metabolite, prior to clustering for better visualization. One minus Pearson’s correlation was used for the similarity metric. A fire color scheme was used in heat maps of the metabolites. Z-score analysis scaled each metabolite according to a reference distribution.

To evaluate the performance of the classification models, these 310 samples were randomly assigned to training (75%) and test (25%) sets. To avoid overfitting, 10-fold cross-validation was performed for models on the training set. The primary statistical learning method used for allograft outcome classification was Random Forests [31] via the randomForest package in R. Significant metabolites were selected from the Random Forests model using the VSURF package in R [32]. Additionally, for visualization of significant metabolites, volcano plots were produced using variable importance values derived from Random Forests models as a significance measure. Metabolite selection was done by Bonferroni-corrected *p*-value in addition to VSURF to display a traditional volcano plot and directly compare VSURF to traditional t-testing methods and their resulting metabolite lists. These variable importance scores are defined as the mean percentage decrease in classification accuracy of the model if the metabolite data were to be randomly permuted rather than taken as quantified (a higher score denotes a higher variable importance). Comparison of classification models was done by computing and plotting area under the curve (AUC) from the receiver operating characteristic (ROC) using the pROC package in R. Statistical comparison between full and abbreviated metabolite models to assess diagnostic accuracy similarity was carried out using the DeLong’s test [33]. Given that certain clinical data variables were significantly different between groups, these variables were reviewed for any association with particular variable differences within or between groups and their impact on metabolite signatures of different transplant phenotypes. Analysis was performed using the R statistical software version 3.4.3. MetaboAnalyst (www.metaboanalyst.ca) was used to perform targeted pathway and enrichment analysis [34].

### 2.4. Data Availability

The datasets generated during and analyzed during the current study are not publicly available due to legacy IRB consent restrictions on public sharing of data from these patient populations but are available from the corresponding author on reasonable request.

## 3. Results

### 3.1. Metabolites in Urine Are Perturbed in Different Transplant Injuries in Kidney Transplantation

**Figure 1:**
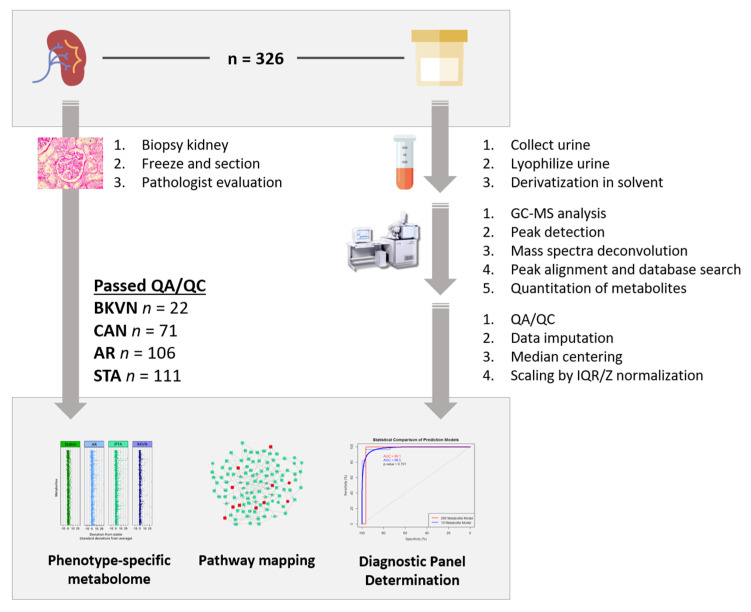
Sample selection and study schematic of the study. Summary outlining study samples, assay platform, study phenotypes, analysis, and results.

We processed 326 urine samples for a targeted metabolomics assay that identified 266 metabolites. Figure 1 summarizes the study. Sixteen samples had missing data on more than one third of total metabolites identified following a tool called MissForest on non-parametric missing value imputation for mixed-type data [30]. Metabolomics data on the remaining 310 biopsy-matched urine samples was used for the analyses of both post-Tx injury detection and associated metabolic pathways and their enrichment. Baseline characteristics of the study subjects is provided in Table 1.

**Figure 2:**
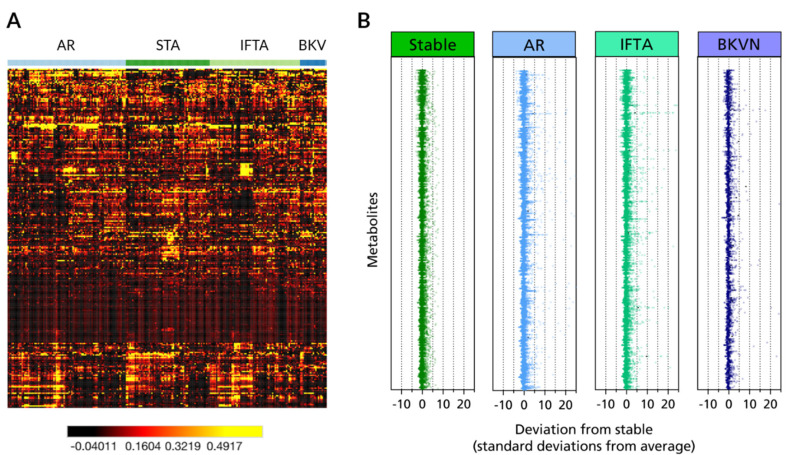
Metabolomic profiling of renal transplant outcomes. (**A**) Heat map representation of unsupervised hierarchical clustering by metabolite (rows) grouped by transplant phenotype (columns). Shades of black to red to orange to yellow represent continuous increases of a metabolite relative to the median metabolite levels (see color scale). (**B**) z-score plots for the data in a normalized to the mean of the stable phenotype urine samples (truncated at 25 s.d. for clarity).

The data was used for supervised clustering to generate a heat map (Figure 2A) and z-score plot (Figure 2B). The heatmap shows heterogeneity in overall metabolome data across urine samples from different phenotypes. In the z-score plot, stable-based z-scores were plotted for each of the 266 metabolites. The plots revealed robust metabolic alterations in AR (z-score range: −4.2 to 800.5) and IFTA (z-score range: −3.8 to 265.4) compared to fewer changes in BKVN samples (z-score range: −3.4 to 116.9).

### 3.2. Metabolite Marker Panel for Alloimmune Injury

**Figure 3:**
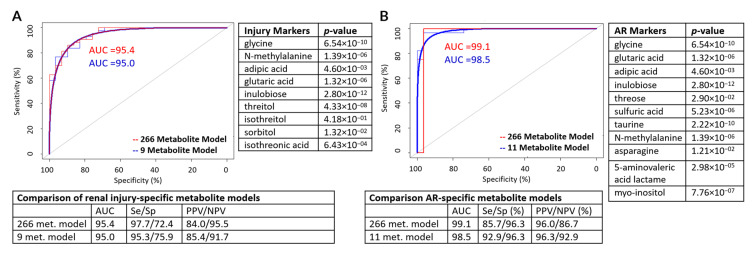
Identification of potential biomarker panel of metabolites for KTx alloimmune injury and acute rejection using VSURF method. (**A**) Two receiver operating characteristic (ROC) curves representing classification accuracies and a statistical comparison of the full and sparse RF models for alloimmune injury and the table displaying classification accuracy on the test set. The metabolites in the panel are listed on the right-hand side (**B**) Two ROC curves representing classification accuracies and a statistical comparison of the full and sparse Random Forests (RF) models for acute rejection (AR) injury and the bottom table displaying classification accuracy on the test set. The metabolites in the panel are listed on the right-hand side.

Applying the VSURF method, a panel of 9 metabolites (Table 2) were selected out of 266 to accurately classify post-Tx alloimmune injury, combining the output from samples with either acute or chronic alloimmune injury (AR/IFTA) versus stable (STA) samples. The resulting model had a 95% accuracy of correctly discriminating between the two outcome groups (AUC = 0.950, sensitivity = 95.3%, specificity = 75.9%). This lower specificity is likely due to within group heterogeneity between AR and IFTA phenotypes. The 9 metabolite VSURF model was nearly identical in accuracy to the full 266-metabolite model, which had an AUC of 0.954. This difference in AUC values was not significant using DeLong’s test (*p* = 0.731), meaning there is no significant change in classification accuracy between the full and abbreviated metabolite models (Figure 3A). This suggests that no diagnostic accuracy is lost in using the abbreviated metabolite model.

### 3.3. Metabolite Marker Panel for Acute Rejection

**Figure 4:**
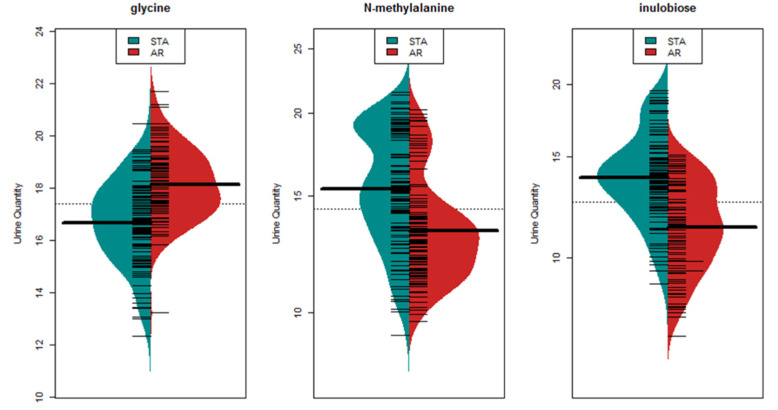
Significantly altered metabolites in AR versus STA. Bean plots demonstrating distribution of the 3 most significant metabolites in AR comparing to STA. The bold horizontal line represents mean value for each group.

In order to identify a metabolite marker panel specific to acute rejection of KTx, we applied VSURF exclusively to the AR and STA urine metabolome datasets (*n* = 217). The resulting model contained 11 metabolites (Table 2) for AR detection. The ROC analysis resulted with an AUC of 0.985 with 92.9% sensitivity and 96.3% specificity (Figure 3B). Individual distributions for the three most significant metabolites, glycine, *N*-methylalanine, and inulobiose, are presented in the form of bean plots (Figure 4).

### 3.4. Metabolite Marker Panel for BK Virus Nephritis

In order to identify BKVN-specific metabolites, we used VSURF on 22 BKVN urine and 288 non-BKVN urine that included AR, IFTA, and STA urine. The resulting VSURF panel contained 5 metabolites, Arabinose, 2-hydroxy-2-methylbutanoic acid, hypoxanthine, benzyl alcohol, and *N*-acetyl-d-mannosamine (Table 2) for BKVN classification with 72.7% sensitivity and 96.2% specificity (Table S1). When we confined our analysis to only BKVN vs. STA, VSURF resulted in a panel of 4 metabolites, arabinose, 2-hydroxy-2-methylbutanoic acid, octadecanol, and phosphate. For this panel, BKVN classification was 88.9% sensitive and 94.8% specific (Table S2). The 4-metabolite VSURF model had accuracy comparable to that of the full 266-metabolite model, which had a sensitivity of 87.5% and specificity of 93.2% (Table S3).

### 3.5. Metabolic Pathways Associated with Graft Injury

**Figure 5:**
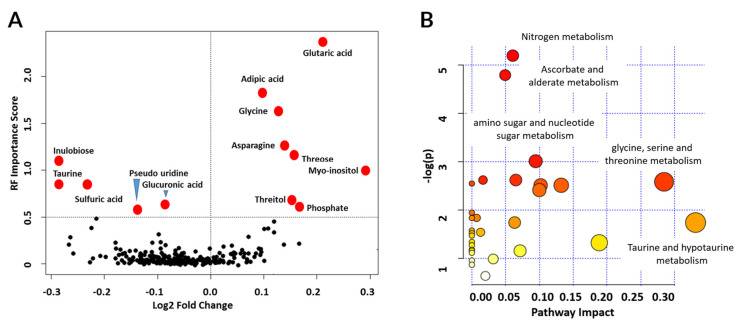
Metabolites and pathways significantly perturbed in KTx alloimmune injury. (**A**) Volcano plot displaying fold change and significance of metabolites. Red dots denote metabolites significant at a Random Forests importance score greater than 0.5. The right half displays metabolites in the injury group with a higher signature relative to the stable group. Some metabolites from the 9-metabolite marker panel for alloimmune injury and the 11-metabolite marker panel for AR are among the very highly perturbed metabolites labeled in red dots. (**B**) Enrichment analysis of metabolic pathways using significantly altered metabolites showed enrichment in nitrogen metabolism (*p* = 0.0055), ascorbate and aldarate metabolism (*p* = 0.0083), and amino sugar and nucleotide sugar metabolism (*p* = 0.05) as significantly enriched pathways. The y-axis represents the *p*-values as the negative of their natural logarithm.

To explore metabolite significance by both statistical significance and magnitude of fold change in the injury group, a volcano plot with Random Forests (RF) importance score was generated (Figure 5A) that shows the relative importance of the metabolite in terms of RF score for AR-specific panel. Additionally, a volcano plot with fold changes (increased or decreased) and corresponding *p*-values displayed the significance of the various metabolites in AR (Figure S1). The plot reveals metabolites of increasing significance relative to the Random Forests classification model. Some metabolites from the 9-metabolite marker panel for alloimmune injury and the 11-metabolite marker panel for AR are among the very highly perturbed metabolites. The metabolites significantly perturbed in KTx injury with *p*-value < 0.001 (*n* = 42) were analyzed for metabolic pathway enrichment with MetaboAnalyst. Pathway analysis for enrichment identified nitrogen metabolism, ascorbate, and aldarate metabolism, and amino sugar and nucleotide sugar metabolism as the three most significantly enriched pathways (Figure 5B).

## 4. Discussion

Sophisticated interrogation of urine through advanced technologies for kidney diseases is important as urine provides an attractive alternative biospecimen [35] and unlike invasive biopsies, urine metabolite changes can be diagnostic of advanced tissue injury. Additionally, our data suggest that these urine panels can have much greater sensitivity and specificity over measured serum creatinine [35,36,37,38,39]. Molecular perturbations in the kidney have been previously shown to occur much earlier than both histological changes and clinical alterations in kidney function, and previously published studies confirm that urine is an excellent mirror of these intra-graft molecular changes [11,35,40,41,42,43,44,45,46,47,48,49]. In this comprehensive study, we had access to a unique resource of over 300 biopsy matched urine samples archived from kidney transplant patients transplanted at multiple transplant centers, mapped with detailed clinical demographics, which enhanced the results obtained from the urine metabolomic studies conducted in this study.

Prior studies, using different assay platforms, have evaluated the urine metabolome for assessing kidney transplant injury [43,50,51,52]. Wang et al. [50] applied matrix-assisted laser desorption/ionization Fourier transform mass spectrometry (MALDI-FTMS) for studying acute tubular injury, Blydt-Hanson et al. [51] used liquid chromatography tandem mass spectrometry for studying transplant rejection, Dieme et al. [52] used GC-MS to study the metabolic effects of calcineurin inhibitor drugs in kidney transplant patients, Ho et al. [53] used LC-MS for analysis of alloimmune injury, and Suhre et al. [43] applied LC-MS and GC-MS. Most of these studies have larger metabolite diagnostic panels and only few studies have urine samples that are all biopsy-matched, resulting in little overlap across the identified panels to date. Using all biopsy-matched urine samples matched with pathologist blinded biopsy evaluations, GC-MS, and custom informatics analyses using nonlinear, nonparametric machine learning model development, we were able to greatly refine, as well as cross-validate the performance of small panels of urine metabolites for AR and BKVN. These clinical phenotypes are often difficult to distinguish even by biopsy, and pose a clinical challenge for patient management, specifically concerning the decision between immunosuppression augmentation (in AR) or minimization (in BKVN). In addition to selecting the most informative metabolites to provide discriminant diagnostic models for biopsy matched transplant injury categories, we have also tried to better understand the biological mechanisms that some of the metabolites suggest are dysregulated in transplant injury, using panels in this study and other published studies on the urine metabolome.

Many of the metabolites that we identified as correlated with transplant injury have previously been associated with changes in renal physiology. Taurine is a key metabolite that was included in our AR specific panel and was found to have significantly reduced levels in urine during rejection. Taurine plays a role in different physiologic and biologic processes in the kidney as reflected in urinary excretion patterns. Taurine participates in several physiological functions in the kidney [54,55,56], such as its role in the renal cell cycle and apoptosis. It also functions as an osmolyte during the stress response. Therefore, the changes in urinary taurine levels seen in this study may relate to the higher burden of tissue injury in AR, and its lower levels in the kidney may reflect a failure in protecting the kidney during immune-mediated damage [57]. The low level of taurine in urine during rejection may reflect a combination of decreased production in the kidney and perturbed osmolar reabsorption of taurine in the medulla [58].

Myo-inositol was found to be a significant biomarker in our model and had been previously shown by Dieme et al. [52] and Suhre et al. [43] to be relevant to and increased in transplant rejection. Urinary myo-inositol was the most important metabolite for discriminating between AR, STA, and IFTA phenotypes in our models. Myo-inositol is an osmolyte of the renal medulla that plays an important role in protecting renal cells from hyperosmotic stress [59]. It is enriched under hyperosmotic conditions via the sodium/myo-inositol cotransporter in the thick ascending limb of the loop of Henle [60]. The kidney is the most important organ for myo-inositol metabolism given that there is high expression of its associated enzymes, l-myo-inositol-1-phosphate synthetase, and myo-inositol oxygenase, in the renal parenchyma [61]. Inhibition of myo-inositol transport has been shown to cause acute renal failure in rats [62]. Furthermore, it has recently been shown through urine metabolomics profiling of humans that increased levels of myo-inositol are significantly associated with kidney disease and inversely proportional to eGFR [63]. It has also been shown to be elevated in the plasma metabolomic profiles of patients with end-stage renal disease [50,61]. These studies suggest an essential role of myo-inositol in renal physiology. Thus, its higher levels in AR may relate to the severity or progression of rejection. In looking at known perturbations in gene expression levels in AR [14] we observed that the sodium-myo-inositol transporter (SMIT), encoded by *SLC5A3*, is located in the thick ascending limb and functions to reabsorb myo-inositol into the renal medullary cells under conditions of hypertonicity [64]. Thus, perturbations of this transporter may be a key mechanism for acute rejection related tissue injury, mediated by hyperosmolar stress [63]. Given that patients with AR may have preserved kidney graft function and stable serum creatinine levels, the utility of this biomarker may be confounded given its correlation with eGFR. Further studies are needed to further validate role of myo-inositol in the progression of rejection.

These are first observations, should be consolidated with more integrative analysis of multi-omic datasets, and could be further aided by spatial metabolomics. Use of metabolomics for kidney disease outcomes generally could complement diagnostics made using other modalities, including gene expression, cell-free DNA, and proteomics [65,66,67,68,69].

This study benefits from the evaluation of the human urine metabolome in different geographic and demographic cohorts, as the samples came from enrolled patients at Stanford and UCSF. The diversity of patient samples supports the robustness and clinical utility of the described metabolite panels. Translation of these biomarker panels to clinical practice can be done by GC-MS/TOF based assays that are readily available in most commercial labs. Despite the fact that we have analyzed urine from a diverse population and using two different immunosuppressive drugs, additional independent validation studies that allow for prospective clinical testing and application of these metabolite panels will be required to validate the performance of these biomarker panels for diagnostic transplant monitoring. This is important because the diversity of the cohort also introduces many factors that are known to affect the metabolic profiles of patients, such as age, gender, BMI, diet, exercise, comorbidities, and even the time of day as a function of the circadian rhythm [70]. In our cohorts, age and gender were significantly different and as would be expected from a pediatric population, they had few comorbidities. A future study consisting of a larger cohort size that collected these additional details would help to delineate the effects of these parameters on the metabolic profiles. Furthermore, we note that storage length is known to affect the metabolites present and detected in urine studies. While our urine samples were stored at −80 °C, this may have influenced the distribution of the metabolites in the samples. Future work should be done to compare biobanked samples versus freshly collected samples to delineate any potential differences in the metabolomic profile.

There are a number of additional limitations to this study. We note that certain patient subsets, such as the BKVN arm had a relatively smaller number of patients (*n* = 22), and this warrants further investigation with a larger number of samples to validate the results. We also note that all samples AR samples used were collected at the time of the rejection, rather than before, and thus we could not assess the predictive value of metabolite alterations in transplant rejection. We believe this would be a useful area of future study, as predictive signatures of rejection prior to clinical AR (e.g., picking up subclinical AR) would be valuable to prevent further decline of kidney function [71].

Nevertheless, urinary metabolite profiles provide an exciting opportunity for rapid bedside–to-bench screening for risk assessment, improved immunosuppression titration, and rejection prevention to ultimately improve transplant and patient outcomes. With the increasing number of studies in this space [43,72], a comprehensive metabolomics picture of allograft outcomes can be created and further contribute to the care management of transplant patients.

## Figures and Tables

**Table 1:** Patient demographic data for the discovery cohort.

Phenotype	AR	STA	IFTA	BKVN	*p*-Value
Number of Patients	106	111	71	22	
Maintenance (% Steroid-free)	63.2%	50.5%	56.3%	36.4%	0.078
Recipient Gender (% M)	64.2%	58.6%	67.6%	59.1%	0.002
Recipient Age * (years)	13 ± 5 (14; 2–21)	14 ± 5 (15; 1–21)	10 ± 6 (10; 1–20)	14 ± 5 (17; 1–18)	0.003
Donor Gender (% M)	46.2%	52.3%	52.1%	72.7%	0.123
Donor Age * (years)	29 ± 11 (29; 4–50)	30 ± 10 (28; 14–51)	30 ± 10 (32; 12–50)	28 ± 10 (29; 16–49)	0.353
Month post-Tx (mean ± SD)	71 ± 32	15 ± 24	23 ± 32	8 ± 7	0.311
Donor Source (%):					
1 = Living Related	1 = 24.5%	1 = 37.8%	1 = 43.7%	1 = 9.1%	
2 = Living Unrelated	2 = 40.6%	2 = 8.1%	2 = 8.5%	2 = 31.8%	
3 = Deceased	3 = 34.0%	3 = 44.1%	3 = 47.9%	3 = 54.5%	
Recipient Race (%):					
1 = Caucasian	1 = 42.5%	1 = 43.2%	1 = 50.7%	1 = 27.3%	
2 = Asian	2 = 5.7%	2 = 4.5%	2 = 7.0%	2 = 0.0%	
3 = African American	3 = 16.0%	3 = 18.0%	3 = 18.3%	3 = 13.6%	
4 = Hispanic	4 = 7.5%	4 = 2.7%	4 = 5.6%	4 = 18.2%	
5 = Mixed and Others	5 = 12.3%	5 = 16.2%	5 = 9.9%	5 = 0.0%	
HLA Mismatch	4.64 ± 1.41	4.15 ± 1.35	3.62 ± 1.67	4.80 ± 1.15	0.245
eGFR	75.3 ± 42.3	95.4 ± 28.5	104.1 ± 36.7	N/A ^#^	0.171

* Age in years: mean ± SD (median; range). AR, acute rejection; STA, stable graft function; IFTA, interstitial fibrosis and tubular atrophy; BKVN, BK virus nephropathy. ^#^ Estimated glomerular filtration rate (eGFR) data were unavailable for BKVN samples. Immunosuppression consisted of Tacrolimus and Mycophenolate Mofetil for all patients, with maintenance steroids for those on steroid-based immunosuppression. All patients received IL2R monoclonal antibody (Daclizumab) induction; steroid-based patients received this for 2 months and steroid-free patients received this for 6 months. Most patients were unsensitized and recipients for first allografts, with 4 repeat transplants. Of the 106 AR, 29 were ABMR. The clinical data variables that were significantly different between groups were assessed for any statistical association with their impact on metabolite signatures of different transplant phenotypes and were not found to be significant.

**Table 2:** Transplant phenotype-specific metabolite markers.

Injury-Specific (*n* = 9)	AR (*n* = 11)	BKVN (*n* = 5)
Glycine	Glycine	Arabinose
*N*-methylalanine	Glutaric acid	2-hydroxy-2-methylbutanoic acid
Adipic acid	Adipic acid	Hypoxanthine
Glutaric acid	Inulobiose	Benzyl alcohol
Inulobiose	Threose	*N*-acetyl-d-mannosamine
Threitol	Sulfuric acid	
Isothreitol	Taurine	
Sorbitol	*N*-methylalanine	
Isothreonic acid	Asparagine	
	5-aminovaleric acid lactam	
	Myo-inositol	

## Supplementary Materials

The following are available online at https://www.mdpi.com/2077-0383/9/8/2341/s1, Table S1: Metabolite selection by VSURF to discriminate BKVN from all other phenotypes, Table S2: Metabolite selection by VSURF to discriminate BKVN from STA, Table S3: Use of all 266 metabolites in VSURF to discriminate BKVN from STA, Figure S1: Volcano plot displaying fold change and significance of metabolites in the urine of acute rejection patients compared to the patients with stable grafts.

## References

[1] Abecassis M., Bartlett S.T., Collins A.J., Davis C.L., Delmonico F.L., Friedewald J.J., Hays R., Howard A., Jones E., Leichtam A.B. (2008). Kidney transplantation as primary therapy for end-stage renal disease: A National Kidney Foundation/Kidney Disease Outcomes Quality Initiative (NKF/KDOQITM) conference. Clin. J. Am. Soc. Nephrol. CJASN.

[2] Pomfret E.A., Sung R.S., Allan J., Kinkhabwala M., Melancon J.K., Roberts J.P. (2008). Solving the organ shortage crisis: The 7th annual American Society of Transplant Surgeons’ State-of-the-Art Winter Symposium. Am. J. Transplant..

[3] Meier-Kriesche H.U., Schold J.D., Srinivas T.R., Kaplan B. (2004). Lack of improvement in renal allograft survival despite a marked decrease in acute rejection rates over the most recent era. Am. J. Transplant..

[4] Gaston R.S. (2016). Improving Long-Term Outcomes in Kidney Transplantation: Towards a New Paradigm of Post-Transplant Care in the United States. Trans. Am. Clin. Climatol. Assoc..

[5] Nasr M., Sigdel T., Sarwal M. (2016). Advances in diagnostics for transplant rejection. Expert Rev. Mol. Diagn..

[6] Loupy A., Haas M., Solez K., Racusen L., Glotz D., Seron D., Nankivell B.J., Colvin R.B., Afrouzian M., Akalin E. (2017). The Banff 2015 Kidney Meeting Report: Current Challenges in Rejection Classification and Prospects for Adopting Molecular Pathology. Am. J. Transplant..

[7] Filler G., Bendrick-Peart J., Christians U. (2008). Pharmacokinetics of mycophenolate mofetil and sirolimus in children. Ther. Drug Monit..

[8] Yang J.Y., Sarwal M.M. (2017). Transplant genetics and genomics. Nat. Rev. Genet..

[9] Reeve J., Bohmig G.A., Eskandary F., Einecke G., Lefaucheur C., Loupy A., Halloran P.F. (2017). The MMDx-Kidney Study Group. Assessing rejection-related disease in kidney transplant biopsies based on archetypal analysis of molecular phenotypes. JCI Insight.

[10] Roedder S., Sigdel T., Salomonis N., Hsieh S., Dai H., Bestard O., Metes D., Zeevi A., Gritsh A., Cheeseman J. (2014). The kSORT assay to detect renal transplant patients at high risk for acute rejection: Results of the multicenter AART study. PLoS Med..

[11] Sigdel T.K., Salomonis N., Nicora C.D., Ryu S., He J., Dinh V., Orton D.J., Moore R.J., Hsieh S.C., Dai H. (2014). The identification of novel potential injury mechanisms and candidate biomarkers in renal allograft rejection by quantitative proteomics. Mol. Cell. Proteom. MCP.

[12] Khatri P., Roedder S., Kimura N., De Vusser K., Morgan A.A., Gong Y., Fischbein M.P., Robbins R.C., Naesens M., Bute A.J. (2013). A common rejection module (CRM) for acute rejection across multiple organs identifies novel therapeutics for organ transplantation. J. Exp. Med..

[13] Yang J.Y., Sigdel T.K., Sarwal M.M. (2016). Self-antigens and rejection: A proteomic analysis. Curr. Opin. Organ Transplant..

[14] Sarwal M., Chua M.S., Kambham N., Hsieh S.C., Satterwhite T., Masek M., Salvatierra O. (2003). Molecular heterogeneity in acute renal allograft rejection identified by DNA microarray profiling. N. Engl. J. Med..

[15] Erpicum P., Hanssen O., Weekers L., Lovinfosse P., Meunier P., Tshibanda L., Ktzesinski J.M., Hustinx R., Jouret F. (2017). Non-invasive approaches in the diagnosis of acute rejection in kidney transplant recipients, part II: Omics analyses of urine and blood samples. Clin. Kidney J..

[16] Wishart D.S. (2008). Metabolomics: A complementary tool in renal transplantation. Contrib. Nephrol..

[17] Klawitter J., Klawitter J., Kushner E., Jonscher K., Bendrick-Peart J., Leibfritz D., Christians U., Schmitz V. (2010). Association of immunosuppressant-induced protein changes in the rat kidney with changes in urine metabolite patterns: A proteo-metabonomic study. J. Proteome Res..

[18] Bouatra S., Aziat F., Mandal R., Guo A.C., Wilson M.R., Knox C., Bjorndahl T.C., Krishamurthy R., Saleem F., Liu P. (2013). The human urine metabolome. PLoS ONE.

[19] Bohra R., Klepacki J., Klawitter J., Klawitter J., Thurman J., Christians U. (2013). Proteomics and metabolomics in renal transplantation-quo vadis?. Transpl. Int..

[20] Gromski P.S., Muhamadali H., Ellis D.I., Xu Y., Correa E., Turner M.L., Goodacre R. (2015). A tutorial review: Metabolomics and partial least squares-discriminant analysis-a marriage of convenience or a shotgun wedding. Anal. Chim. Acta.

[21] Racusen L.C., Halloran P.F., Solez K. (2004). Banff 2003 meeting report: New diagnostic insights and standards. Am. J. Transplant..

[22] Racusen L.C., Solez K., Colvin R.B., Bonsib S.M., Castro M.C., Cavallo T., Croker B.P., Demetris A.J., Drachenberg C.B., Fogo A.B. (1999). The Banff 97 working classification of renal allograft pathology. Kidney Int..

[23] Solez K., Colvin R.B., Racusen L.C., Haas M., Sis B., Mengel M., Halloran P.F., Baldwin W., Banfi G., Collins A.B. (2008). Banff 07 classification of renal allograft pathology: Updates and future directions. Am. J. Transplant..

[24] Nankivell B.J., Alexander S.I. (2010). Rejection of the kidney allograft. N. Engl. J. Med..

[25] Fletcher J.T., Nankivell B.J., Alexander S.I. (2009). Chronic allograft nephropathy. Pediatric Nephrol..

[26] Kind T., Wohlgemuth G., Lee D.Y., Lu Y., Palazoglu M., Shahbaz S., Fiehn O. (2009). FiehnLib: Mass spectral and retention index libraries for metabolomics based on quadrupole and time-of-flight gas chromatography/mass spectrometry. Anal. Chem..

[27] Kind T., Tolstikov V., Fiehn O., Weiss R.H. (2007). A comprehensive urinary metabolomic approach for identifying kidney cancerr. Anal. Biochem..

[28] Katajamaa M., Miettinen J., Oresic M. (2006). MZmine: Toolbox for processing and visualization of mass spectrometry based molecular profile data. Bioinformatics.

[29] Smith C.A., Want E.J., O’Maille G., Abagyan R., Siuzdak G. (2006). XCMS: Processing mass spectrometry data for metabolite profiling using nonlinear peak alignment, matching, and identification. Anal. Chem..

[30] Stekhoven D.J., Buhlmann P. (2012). MissForest-non-parametric missing value imputation for mixed-type data. Bioinformatics.

[31] Breiman L. (2001). Random forests. Mach. Learn..

[32] Genuer R., Poggi J.M., Tuleau-Malot C. (2015). VSURF: An R Package for Variable Selection Using Random Forests. R. J..

[33] Delong E.R., Delong D.M., Clarkepearson D.I. (1988). Comparing the Areas under 2 or More Correlated Receiver Operating Characteristic Curves-a Nonparametric Approach. Biometrics.

[34] Chong J., Soufan O., Li C., Caraus I., Li S., Bourque G., Wishart D.S., Xia J. (2018). MetaboAnalyst 4.0: Towards more transparent and integrative metabolomics analysis. Nucleic Acids Res..

[35] Nissaisorakarn V., Lee J.R., Lubetzky M., Suthanthiran M. (2018). Urine biomarkers informative of human kidney allograft rejection and tolerance. Hum. Immunol..

[36] Sigdel T.K., Kaushal A., Gritsenko M., Norbeck A.D., Qian W.J., Xiao W., Camp D.G., Smith R.D., Sarwal M.M. (2010). Shotgun proteomics identifies proteins specific for acute renal transplant rejection. Proteom. Clin. Appl..

[37] Sigdel T.K., Lee S., Sarwal M.M. (2011). Profiling the proteome in renal transplantation. Proteom. Clin. Appl..

[38] Sigdel T.K., Sarwal M.M. (2008). The proteogenomic path towards biomarker discovery. Pediatric Transplant..

[39] Sigdel T.K., Sarwal M.M. (2011). Recent advances in biomarker discovery in solid organ transplant by proteomics. Expert Rev. Proteom..

[40] Sigdel T.K., Gao Y., He J., Wang A., Nicora C.D., Fillmore T.L., Shi T., Webb-Robertson B.J., Smith R.D., Qian W.J. (2016). Mining the human urine proteome for monitoring renal transplant injury. Kidney Int..

[41] Sigdel T.K., Ng Y.W., Lee S., Nicora C.D., Qian W.J., Smith R.D., Qian W.J., Salvatierra O., Camp D.G., Sarwal M.M. (2014). Perturbations in the urinary exosome in transplant rejection. Front. Med..

[42] Sigdel T.K., Vitalone M.J., Tran T.Q., Dai H., Hsieh S.C., Salvatierra O., Sarwal M.M. (2013). A rapid noninvasive assay for the detection of renal transplant injury. Transplantation.

[43] Suhre K., Schwartz J.E., Sharma V.K., Chen Q., Lee J.R., Muthukumar T., Dadhania D.M., Ding R., Ikle D.H., Bridges N.D. (2016). Urine Metabolite Profiles Predictive of Human Kidney Allograft Status. J. Am. Soc. Nephrol. JASN.

[44] Fairchild R.L., Suthanthiran M. (2015). Urine CXCL10/IP-10 Fingers Ongoing Antibody-Mediated Kidney Graft Rejection. J. Am. Soc. Nephrol. JASN.

[45] Li B., Hartono C., Ding R., Sharma V.K., Ramaswamy R., Qian B., Serur D., Mouradian J., Schwartz J.E., Suthanthiran M. (2001). Noninvasive diagnosis of renal-allograft rejection by measurement of messenger RNA for perforin and granzyme B in urine. N. Engl. J. Med..

[46] Li R., Guo L.X., Li Y., Chang W.Q., Liu J.Q., Liu L.F., Xin G.Z. (2017). Dose-response characteristics of Clematis triterpenoid saponins and clematichinenoside AR in rheumatoid arthritis rats by liquid chromatography/mass spectrometry-based serum and urine metabolomics. J. Pharm. Biomed. Anal..

[47] Schaub S., Mayr M., Honger G., Bestland J., Steiger J., Regeniter A., Mihatsch M.J., Wilkins J.A., Rush D., Nickerson P. (2007). Detection of subclinical tubular injury after renal transplantation: Comparison of urine protein analysis with allograft histopathology. Transplantation.

[48] Schaub S., Rush D., Wilkins J., Gibson I.W., Weiler T., Sangster K., Nicolle L., Karpinski M., Jeffery J., Nickerson P. (2004). Proteomic-based detection of urine proteins associated with acute renal allograft rejection. J. Am. Soc. Nephrol. JASN.

[49] Torng S., Rigatto C., Rush D.N., Nickerson P., Jeffery J.R. (2001). The urine protein to creatinine ratio (P/C) as a predictor of 24-hour urine protein excretion in renal transplant patients. Transplantation.

[50] Choi J.Y., Yoon Y.J., Choi H.J., Park S.H., Kim C.D., Kim I.S., Kwon T.H., Do J.Y., Kim S.H., Ryu D.H. (2011). Dialysis modality-dependent changes in serum metabolites: Accumulation of inosine and hypoxanthine in patients on haemodialysis. Nephrol. Dial. Transpl..

[51] Blydt-Hansen T.D., Sharma A., Gibson I.W., Mandal R., Wishart D.S. (2014). Urinary metabolomics for noninvasive detection of borderline and acute T cell-mediated rejection in children after kidney transplantation. Am. J. Transplant..

[52] Dieme B., Halimi J.M., Emond P., Buchler M., Nadal-Desbarat L., Blasco H., Guellec C.L. (2014). Assessing the metabolic effects of calcineurin inhibitors in renal transplant recipients by urine metabolic profiling. Transplantation.

[53] Ho J., Sharma A., Mandal R., Wishart D.S., Wiebe C., Storsley L., Karpinski M., Gibson I.M., Nickerson P.W., Rush D.N. (2016). Detecting Renal Allograft Inflammation Using Quantitative Urine Metabolomics and CXCL10. Transplant. Direct.

[54] Hoffman N.E., Iser J.H., Smallwood R.A. (1975). Hepatic bile acid transport: Effect of conjugation and position of hydroxyl groups. Am. J. Physiol.

[55] Chesney R.W., Han X., Patters A.B. (2010). Taurine and the renal system. J. Biomed. Sci..

[56] Trachtman H., Sturman J.A. (1996). Taurine: A therapeutic agent in experimental kidney disease. Amino Acids.

[57] Trachtman H., Futterweit S., Prenner J., Hanon S. (1994). Antioxidants reverse the antiproliferative effect of high glucose and advanced glycosylation end products in cultured rat mesangial cells. Biochem. Biophys. Res. Commun..

[58] Dantzler W.H., Silbernagl S. (1976). Renal tubular reabsorption of taurine, gamma-aminobutyric acid (GABA) and beta-alanine studied by continuous microperfusion. Pflug. Arch..

[59] Brocker C., Thompson D.C., Vasiliou V. (2012). The role of hyperosmotic stress in inflammation and disease. Biomol. Concepts.

[60] Yorek M.A., Dunlap J.A., Lowe W.L. (1999). Osmotic regulation of the Na+/myo-inositol cotransporter and postinduction normalization. Kidney Int..

[61] Niewczas M.A., Sirich T.L., Mathew A.V., Skupien J., Mohney R.P., Warram J.H., Smiles A., Huang X., Walker W., Byun J. (2014). Uremic solutes and risk of end-stage renal disease in type 2 diabetes: Metabolomic study. Kidney Int..

[62] Kitamura H., Yamauchi A., Sugiura T., Matsuoka Y., Horio M., Tohyama M., Shimada S., Imai E., Hori M. (1998). Inhibition of myo-inositol transport causes acute renal failure with selective medullary injury in the rat. Kidney Int..

[63] Gil R.B., Ortiz A., Sanchez-Nino M.D., Markoska K., Schepers E., Vanholder R., Glorieux G., Schmitt-Kopplin P., Heinzmann S.S. (2018). Increased urinary osmolyte excretion indicates chronic kidney disease severity and progression rate. Nephrol. Dial. Transplant..

[64] Burg M.B., Ferraris J.D. (2008). Intracellular organic osmolytes: Function and regulation. J. Biol. Chem..

[65] Yang J.Y.C., Sarwal R., Ky K., Dong V., Stoller M., Sarwal M., Chi T. (2020). Non-Radiologic Assessment of Kidney Stones by KIT, a Spot Urine Assay. Br. J. Urol. Int..

[66] Yang J.Y.C., Sarwal R.D., Fervenza F.C., Sarwal M.M., Lafayette R.A. (2019). Noninvasive Urinary Monitoring of Progression in IgA Nephropathy. Int. J. Mol. Sci..

[67] Sigdel T.K., Yang J.Y.C., Bestard O., Schroeder A., Hsieh S.-C., Liberto J.M., Qamm I., Geraedts A.C.M., Sarwal M.M. (2019). A urinary Common Rejection Module (uCRM) score for non-invasive kidney transplant monitoring. PLoS ONE.

[68] Sigdel T., Yang J., Bestard O., Hsieh S., Roedder S., Damm I., Liberto J., Nandoe S., Sarwal M. (2017). A Non-Invasive Urinary Common Rejection Module (uCRM) Gene Expression Score Quantifies and Differentiates Kidney Transplant Injury. Am. J. Transplant..

[69] Yang J.Y.C., Sarwal R.D., Sigdel T.K., Damm I., Rosenbaum B., Liberto J.M., Chan-on C., Arreola-Guerra J.M., Alberu J.M., Vincenti F. (2020). A urine score for noninvasive accurate diagnosis and prediction of kidney transplant rejection. Sci. Transl. Med..

[70] Bi H., Guo Z., Jia X., Liu H., Ma L., Xue L. (2020). The key points in the pre-analytical procedures of blood and urine samples in metabolomics studies. Metabolomics.

[71] Moreso F., Carrera M., Goma M., Hueso M., Sellares J., Martorell J., Grinyó J.M., Serón D. (2012). Early subclinical rejection as a risk factor for late chronic humoral rejection. Transplantation.

[72] Bassi R., Niewczas M.A., Biancone L., Bussolino S., Merugumala S., Tezza S., D’Addio F., Nasr M.B., Valderrama-Vasquez A., Usuelli V. (2017). Metabolomic profiling in individuals with a failing kidney allograft. PLoS ONE.

