# Supplementary material for: Targeted Urine Metabolomics for Monitoring Renal Allograft Injury and Immunosuppression in Pediatric Patients
Source: J Clin Med. 2020 Jul 22;9(8):2341. doi: 10.3390/jcm9082341 (PMC7465632; doi:10.3390/jcm9082341)

Supplemental Table S1: Metabolite selection by VSURF to discriminate BKVN from all other phenotypes. VSURF selected the following 5 metabolites to discriminate between BK and non-BK: Arabinose, 2-hydroxy-2-methylbutanoic acid, hypoxanthine, benzylalcohol, and N-acetyl-D-mannosamine to predict BKVN from a pool of samples containing BKVN and non-BKVN (AR, CAN, and STA) using all 266 metabolites.

**Predicting BK vs Non-BK from 5 VSURF-selected metabolites**			
Prediction	BKVN	AR, STA, CAN	Total
RF(+)	16	6	22
RF(-)	6	275	281
Total	22	286	303
Sensitivity=72.7%; Specificity=96.2%;<br>PPV=72.7%%; NPV=97.9%%; acc=96.0%			

Supplemental Table S2: Metabolite selection by VSURF to discriminate BKVN from STA. VSURF selected 4 metabolites: arabinose, 2-hydroxy-2-methylbutanoic acid, octadecanol, and phosphate to predict BKVN from a pool of samples containing BKVN and STA. This subset of predictors improved the prediction accuracy of the model.

**Predicting BK vs. STA from 4 VSURF-selected metabolites**			
Prediction	BKVN	STA	Total
RF(+)	16	6	22
RF(-)	2	109	111
Total	18	115	133
Sensitivity=88.9%; Specificity=94.8%; PPV=72.7%%; NPV=98.2%%; acc=94.0%			

Supplemental Table S3: Use of all 266 metabolites in VSURF to discriminate BKVN from STA. VSURF scores to predict BKVN from a pool of samples containing BKVN and non-BKVN (AR, CAN, and STA) using all 266 metabolites.

**Predicting BK vs Non-BK from All 266 Metabolites**			
Prediction	BKVN	AR, STA, CAN	Total
RF(+)	13	9	22
RF(-)	1	277	278
Total	14	286	300
Sensitivity=92.9%; Specificity=96.9%; PPV=59.1%%; NPV=99.6%%; acc=96.7%			

Supplemental Figure S1. Volcano plot displaying fold change and significance of metabolites in the urine of acute rejection patients compared to the patients with stable grafts. Red dots denote metabolites significant at the Bonferroni-adjusted level. The right half displays urine metabolites increased in the acute rejection relative to the stable grafts.

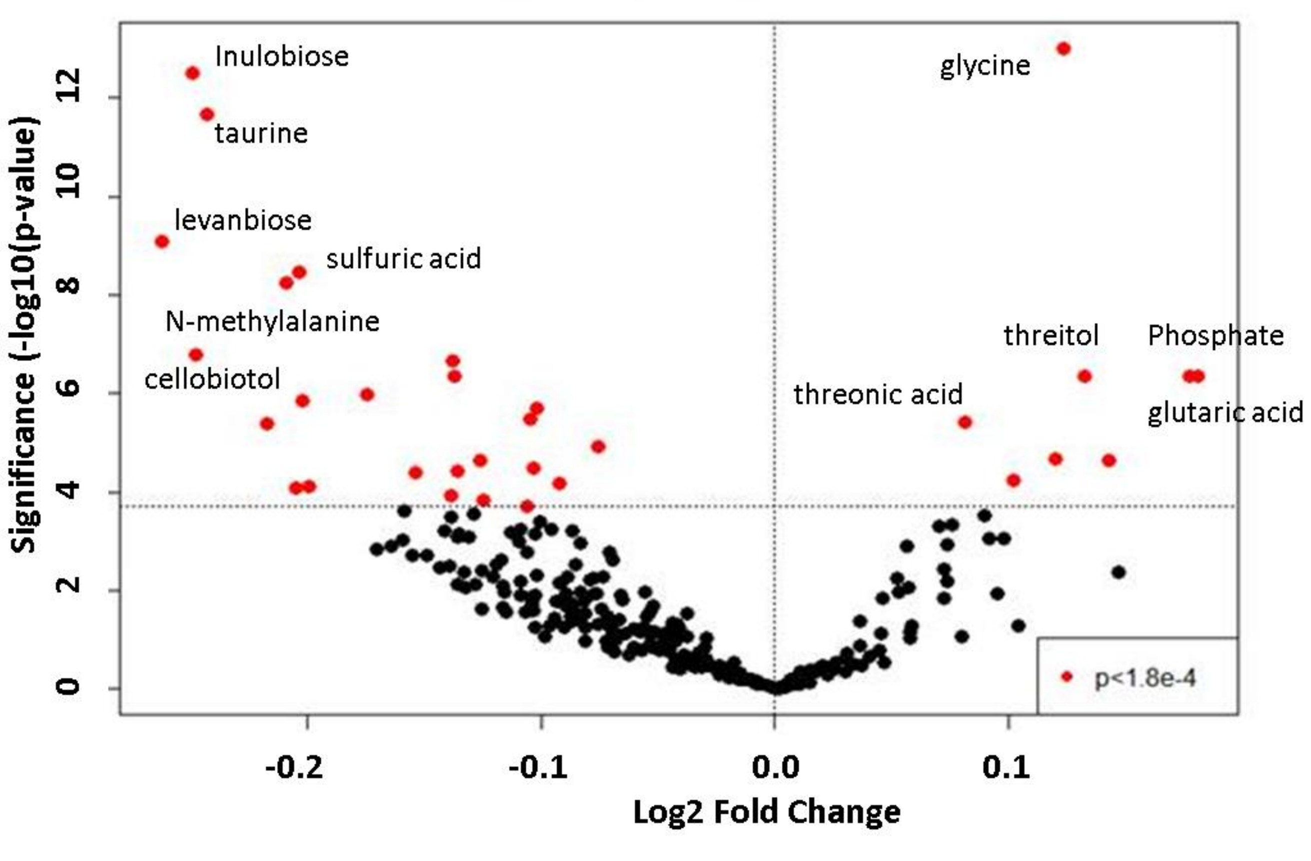

Supplement: Supplementary file 1 [file jcm-09-02341-s001.pdf]
